# Detection of Steel Fatigue Cracks with Strain Sensing Sheets Based on Large Area Electronics

**DOI:** 10.3390/s150408088

**Published:** 2015-04-07

**Authors:** Yao Yao, Branko Glisic

**Affiliations:** Department of Civil and Environmental Engineering, Princeton University, Princeton, NJ 08544, USA; E-Mail: bglisic@princeton.edu

**Keywords:** Structural Health Monitoring (SHM), strain sensing sheet, Large Area Electronics (LAE), direct sensing, fatigue crack, damage detection and localization

## Abstract

Reliable early-stage damage detection requires continuous monitoring over large areas of structure, and with sensors of high spatial resolution. Technologies based on Large Area Electronics (LAE) can enable direct sensing and can be scaled to the level required for Structural Health Monitoring (SHM) of civil structures and infrastructure. Sensing sheets based on LAE contain dense arrangements of thin-film strain sensors, associated electronics and various control circuits deposited and integrated on a flexible polyimide substrate that can cover large areas of structures. This paper presents the development stage of a prototype strain sensing sheet based on LAE for crack detection and localization. Two types of sensing-sheet arrangements with size 6 × 6 inch (152 × 152 mm) were designed and manufactured, one with a very dense arrangement of sensors and the other with a less dense arrangement of sensors. The sensing sheets were bonded to steel plates, which had a notch on the boundary, so the fatigue cracks could be generated under cyclic loading. The sensors within the sensing sheet that were close to the notch tip successfully detected the initialization of fatigue crack and localized the damage on the plate. The sensors that were away from the crack successfully detected the propagation of fatigue cracks based on the time history of the measured strain. The results of the tests have validated the general principles of the proposed sensing sheets for crack detection and identified advantages and challenges of the two tested designs.

## 1. Introduction

### 1.1. Direct and Indirect Sensing

Civil structures and infrastructure in use are aging, deteriorating and eventually approaching their intended service life limit. Money for replacement and repair is scarce and inspection, maintenance, and monitoring play a vital role in attempt to keep these critical structures in safe operation. Structural Health Monitoring (SHM) is defined as the process of implementing strategies and systems for structural damage identification [1]. This process consists of permanent, continuous, periodic, or periodically continuous monitoring of structural parameters, and analysis of recorded data to derive conclusions about structural health, performance and integrity. SHM can provide information aimed at increasing the safety of civil structures and infrastructure, providing support for maintenance decisions and actions, assisting with inspection after natural or manmade disasters, and ultimately creating more resilient structures and cities.

In general, there are two main approaches for damage detection at local scale: (1) direct sensing; and (2) indirect sensing [2]. The direct sensing approach is based on measurements made by sensors or sensing media that are in direct contact with the damage (e.g., strain sensors at the damage location, wave propagation through the damage, *etc.*), and thus the damage is detected and localized directly as an unusual (‘noticeably high’) change in the output of the sensors affected by the damage or as an unusual (‘noticeably important’) alteration of sensing media. The advantage of this approach is its very high reliability in damage detection, localization, and quantification [3,4]; however, the challenge is that this approach requires a large amount of densely placed sensors, which can increase the overall cost and complexity of SHM. The indirect damage detection approach is based on measurements made by sensors that are not in direct contact with damage (e.g., accelerometers, sparsely placed strain sensors that are not at location of the damage, *etc.*). The recorded data is analyzed using various classes of algorithms in order to ascertain damage detection and perform damage characterization (localization and quantification). The advantage of this approach is its use of a relatively small number of sensors. However, the main challenge with indirect sensing is the need for sophisticated data-analysis algorithms, the reliability of which is challenged by noise introduced by environmental influences and loading uncertainties [5].

The direct and indirect sensing approaches have both advantages and challenges (detailed analysis and overview are given in [6]); however, the key advantage of direct sensing approach is its potential for extremely high robustness and reliability of damage detection and localization in real-life settings. While the main challenge of direct sensing approach is the cost, with the development of new sensing technologies, low-cost direct sensing has the potential to play an important role in damage detection and characterization. Consequently, the overall objective of the research performed at SHM*lab* at Princeton University is to enable a pervasive, reliable, and affordable SHM solution based on the direct sensing approach. The main research hypothesis is that by substantially increasing the sensor exposure to local anomalies, direct sensing could lead to greater robustness in damage characterization than current state-of-the-art approaches. The work presented in this paper represents an important step towards the achievement of the above stated objective.

### 1.2. Strain-Based Direct Sensing

Construction material fails at a point when the stress at that point exceeds the strength of the material; however, there are no practical means to directly monitor the stress in real-life settings. Strain is a parameter directly correlated to stress, so any change in the stress field is reflected through a change in the strain field. Hence, the first signs of damage to a structure often have local character and occur in the form of strain-field anomalies. Typical examples are cracks and bowing in steel (which are early indicators of fatigue and loss of local stability), as well as non-structural cracks in concrete (which are early indicators of damage caused by frost, alkali-reactions, or corrosion in reinforced bars). These are reasons to adopt strain as the parameter of interest in this research.

**Figure 1:**
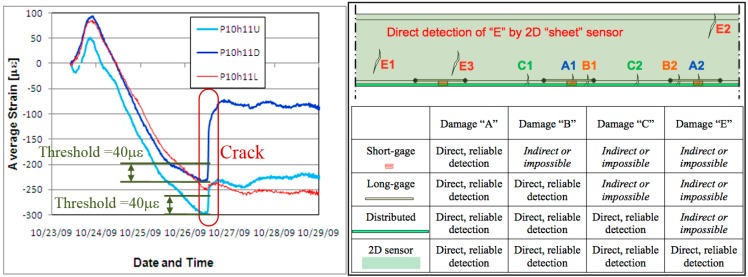
(**Left)**: example of direct damage (crack) detection; (**Right**): schematic comparison between damage detection capabilities of short-gauge, long-gauge, and 1D distributed sensors, and the need for 2D sensing sheets, with permission from [3] (figure does not refer to real case).

Strain sensors which are in direct contact with the strain-field anomaly detect it reliably as an unusually high change in output signal. An example from a real bridge (Streicker Bridge on the Princeton University campus [2]) is shown in Figure 1, left. Three long-gauge fiber-optic sensors (labeled P10h11U, P10h11D, and P10h11L) were embedded in the cross-section of the deck during the pouring of concrete. An early age crack occurred at the locations of P10h11U and P10h11D. As shown in the figure, these sensors were directly activated, creating anomaly signals that can be reliably identified (the thresholds used for detection are annotated in the figure for reference). The sensor P10h11L, however, which was installed less than one meter away, did not cross the crack. Although it recorded a small change (as shown), such minute perturbations are extremely difficult to diagnose, since their magnitude is comparable with changes caused by temperature variations. The error limit of the monitoring system was 4 microstrain, but the tiny crack (<0.1 mm) would be detected by the affected sensors (P10h11U and P10h11D) even if the accuracy was an order of magnitude poorer. This example, from a real application, demonstrates the potential advantages of direct sensing not only for reliable damage detection, but also for robustness against practical interferences.

At present, three categories of strain sensors are commercially available [3]: discrete short-gauge sensors, discrete long-gauge sensors, and continuous (1D) distributed sensors (or sensing cables). A schematic comparison between the damage detection capabilities of these three types of strain sensors based on their spatial disposition is given in Figure 1, right.

Based on the fact that the sensor which is in direct contact with damage would detect it reliably (e.g., [4,7]), Figure 1, right, shows that for commercial short-gauge, long-gauge and 1D distributed sensors the reliability in damage detection increases as the spatial resolution of sensors increases. However, none of the commercially available sensors would be able to reliably detect the damage of type “E” (see Figure 1, right), which motivates and illustrates the need for two-dimensional (2D) distributed sensors. This need was also identified by other researchers, and various technologies have been researched to achieve direct, 2D distributed sensing. These include self-sensing cementitious materials [8], various types of sensing skins based on nano-materials [9,10,11,12], nano-paints [13], conductive polymers [14], photonics crystals [15], and expandable electronics [16].

Other viable options for direct sensing of crack damage include piezoelectric [17] and acoustic [18] techniques that are based on the analysis of wave propagation in the structure. By using active sensors (that serve as wave emitters and receivers) and by performing the appropriate analysis, it is possible to detect and localize cracks. The advantage of these techniques is that they require fewer sensors. The challenges are related to energy requirements and the complexity of the algorithms needed for reliable and accurate damage identification in real-life settings, due to the large size, complex geometrical properties, and variability in the material properties of civil structures (most notably concrete). A comprehensive overview can be found in [6].

### 1.3. Objectives and Scope of the Research

The general objective of this research is to develop and evaluate a prototype of a novel sensing sheet that is inexpensive, easy to manufacture and deploy, and provides densely spaced quantitative measurements from large areas of a structure. The sensing sheet is based on technology called Large Area Electronics (LAE) and consists of dense arrays of sensors and a variety of electronic components (interconnects, circuits, batteries, *etc.*) that are patterned or laminated on a polyimide substrate. In general, the sensors that can be incorporated in this sheet are, for example, strain, pressure, temperature and humidity sensors, and piezoelectric elements. The research presented in this paper represents a stage in reaching the general objective and its scope is limited to strain sensors, to address the challenges related to direct sensing identified in Section 1.1 and Section 1.2. The goal of this paper is to prove the concept of sensing sheet through the tests and assess the performance of two different designs, first with a dense arrangement of sensors and second with less dense arrangement.

This paper first introduces the concept of strain sensing sheet based on large area electronics, and then it describes the manufacturing of sensing sheet samples for test, in which the prototype sensing sheets were bonded to the steel plates. Finally, fatigue tests were carried out on the steel plates to create fatigue cracking, and corresponding results are presented and discussed. The results demonstrate the feasibility of sensing sheets for damage detection over large areas of structures. While the supporting electronics have been proven in the laboratory [19], only the sensors were laminated over the substrate in order to reduce the cost of the test. To further reduce the costs, commercially available full-bridge strain gauges were used and combined with multi-channel reading unit.

## 2. Sensing Sheet Based on Large Area Electronics (LAE)

LAE is a recently developed technology, which enables integration of a broad range of electronic devices onto low-cost thin plastic substrates [20,21]. Using micro-fabrication techniques, several types of thin-film sensors have been demonstrated, including strain sensors [19], pressure sensors [22], vapor sensors [23], particle sensors [24], *etc.* These sensors can be patterned in form of dense arrays that can span large areas (*i.e.*, tens of square meters), and consequently, LAE can potentially provide a novel tool for damage detection and characterization in civil structures and infrastructure [25].

**Figure 2:**
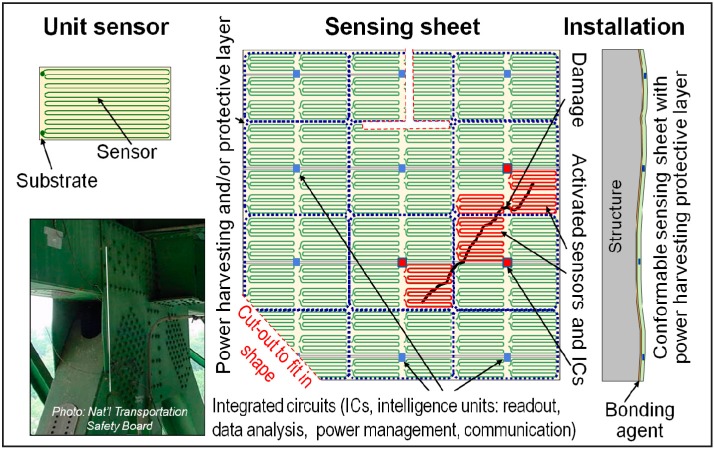
Schematic representation of the sensing sheet based on LAE and ICs, with permission from [26].

Sensing sheets consist of the following: (1) a two-dimensional dense array of unit strain sensors patterned on a polyimide (Kapton) substrate and combined with functional LAE; (2) embedded ICs interfaced via non-contact links for sensor readout, data analysis, power management, and communication; and (3) an integrated flexible photovoltaic sheet and power converters (rechargeable batteries) on the LAE sheet to power the full system and protect it from elements (wind, rain, snow, ultraviolet radiation, *etc.*) [19]. Hence, the strain sensing can be considered as a two-dimensional (2D) quasi-distributed sensor. The concept of a strain sensing sheet and its application are schematically presented in Figure 2.

Damage that occurs in form of a strain field anomaly (irregular black line in Figure 2) will activate the sensors that are in direct contact with it (red-colored sensors in Figure 2), which will measure the unusually high strain change. Thus, it is important to highlight that the purpose of strain sensors is not to actually measure the strain value, but rather to register unusually high strain changes as an indicator of damage. This strain change is detected by the corresponding ICs (red-colored in Figure 2), which will analyze the data and generate the information on damage location and extent based on geographical coordinates of activated sensors. Thus, the sensing sheet will provide detection, localization and information regarding geographical extent of the damage.

**Figure 3:**
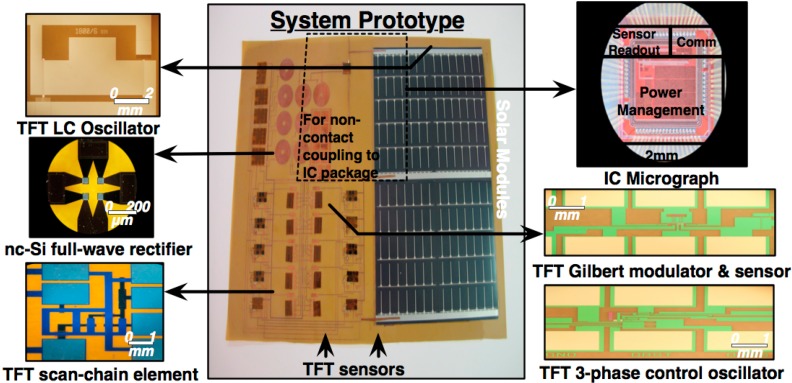
Prototype of sensing sheet for the SHM application (courtesy of Naveen Verma and Yinzhe Hu [28]).

The electronic components of sensing sheet have been researched and developed by a group of researchers at department of electrical engineering of Princeton University. Several components are successfully developed and tested including the sensing sheet system and subsystems architecture, interfacing between the ICs and sensors, scanning circuits, power harvesting and management components, and communication components and protocols. A sensing sheet prototype that includes all developed electronics components is shown in Figure 3. Detailed presentation of the research on the electronics components of sensing sheet is out of the scope of the paper. Interested readers can find more details in published papers [19,27,28].

## 3. Manufacturing and Preparation of Sensing Sheet Samples

**Figure 4:**
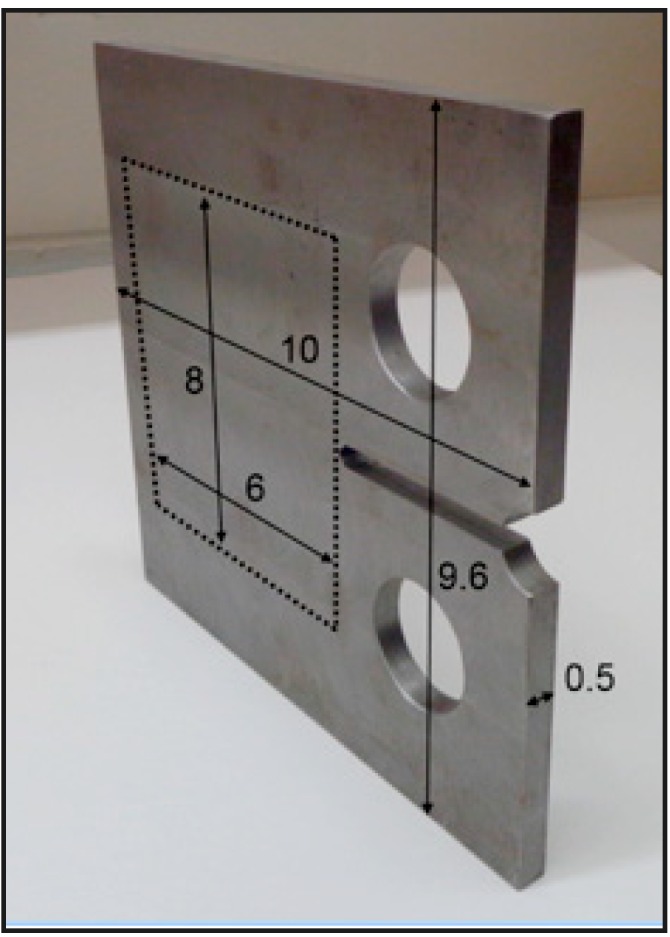
Photograph of the steel test specimen. The area within the dotted line represents the location of the sensing sheet. Dimensions in inch (1 inch = 25.4 mm).

The tests involved application of cyclic load on steel plates, which result in fatigue cracking. The steel plates had a notch in order to predetermine the point of crack occurrence (at the tip of the notch), as shown in Figure 4. The size of the sensing sheet prototype was determined based on the dimensions of steel plates (area delimited with dashed lines in Figure 4).

Manufacturing of the sensing sheet electronic components and integration of ICs as shown in Figure 3 is expensive at the prototype stage. Therefore, to reduce the cost of the tests presented in this paper, commercially available full-bridge strain sensors and associated multi-channel reading units were used. It was judged that this simplification would not affect the validity of the conclusions resulting from the tests, as only mechanical testing was performed. The strain gauges were laminated onto the interconnect, whose purposes were: (1) to provide with physical support to sensors and (2) to provide electrical conductors that guide the signals from the reading unit to sensors and back. Hence, the interconnect consists of a system of conductors patterned over polyimide (Kapton) substrate.

**Figure 5:**
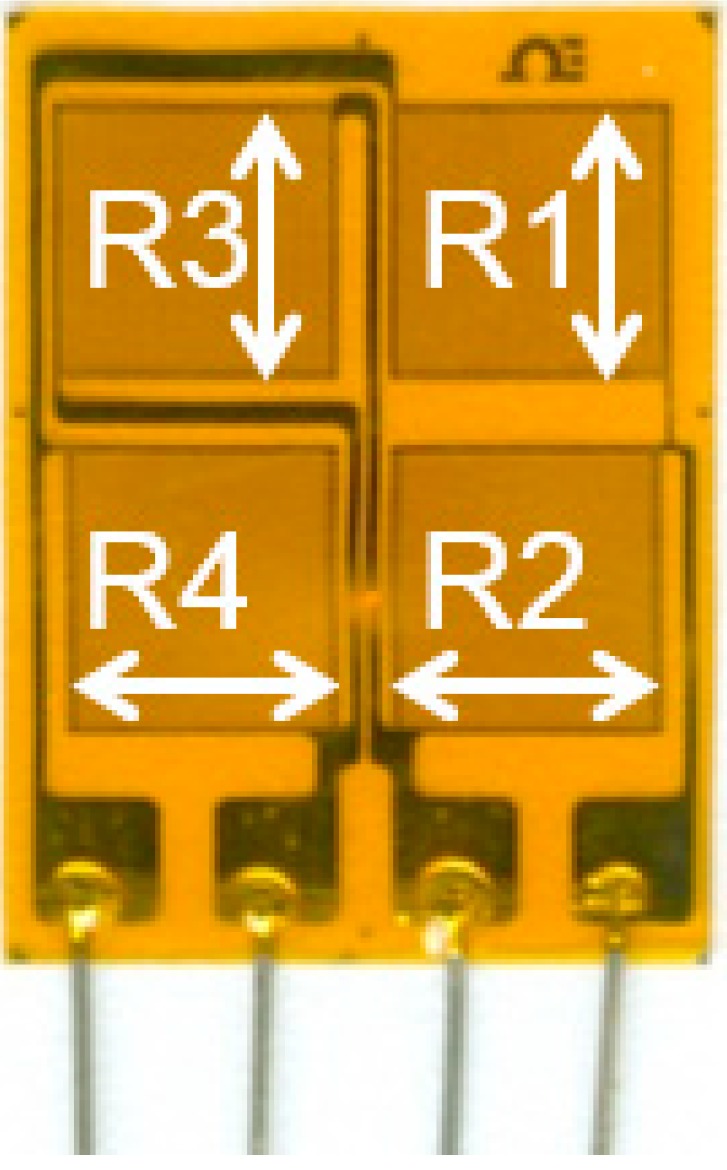
Unit full-bridge sensor (arrows indicate orientation of resistors).

**Figure 6:**
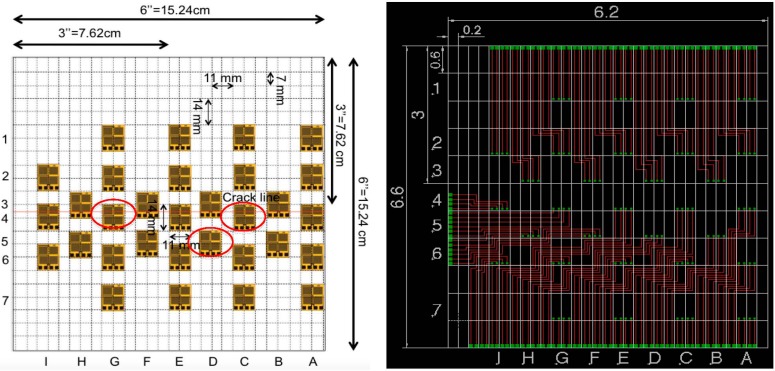
(**Left**): Scheme of sensing sheet Design 1; (**Right**): Corresponding EAGLE PCB Design.

**Figure 7:**
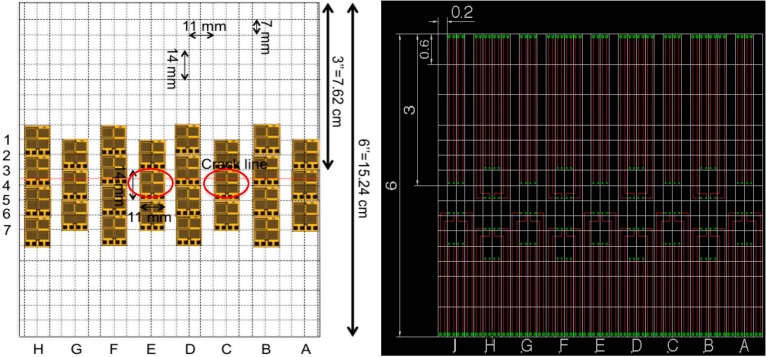
(**Left**): Scheme of sensing sheet Design 2; (**Right**): Corresponding EAGLE PCB Design.

The sensor used in sensing sheet is a full-bridge resistive strain sensor. It consists of four resistors oriented in two mutually perpendicular directions, as shown in Figure 5. Such a sensor provides a differential strain signal, which significantly improves robustness against external influences (e.g., temperature variations). Each resistor is sensitive to strain in the direction of its orientation (indicated by arrows in the figure). More details on the functioning of individual sensor and its behavior under the crack are given in reference [26]. In the sensing sheets, the resistors R1 and R3 are oriented perpendicular to the potential crack propagation line, as shown in Figure 6 and Figure 7.

Preliminary tests demonstrated that the most unfavorable orientation of the sensor with respect to principal strain direction would be diagonal [26], *i.e.*, along diagonals R1-R4 or R2-R3, see Figure 5. In that case, the sensor would measure a value close to zero in a non-damaged structure. In the tests, the resistors R1 and R3 were oriented in the direction of the principal strain in order to ease the interpretation of data. The results of the test presented in Section 5 demonstrate that for damage detection purposes, it is not necessary to know *a priori* the direction of the principal strain (and consequently the orientation of the crack). Two main reasons are: (1) when intersected by a crack, the sensor installed on steel fails almost immediately, regardless of the position (orientation) of the sensors with respect to the crack (*i.e.*, even for unfavorable diagonal orientation), and this failure is actually used to detect the crack; (2) if the crack is in an unfavorable diagonal direction, but it does not intersect any sensor, it will be detected indirectly by the sensors that are the closest to the crack tip (within 10–20 mm), as the direction of principal strain will be changed at that location by the crack occurrence.

Design of the interconnections is an important step toward creation of the sensing sheet. In total two arrangements of individual sensors for the sensing sheets were considered. Figure 6 shows the scheme and corresponding Printed Circuit Board (PCB) diagram of the interconnect for Design 1. Figure 7 shows similar images of Design 2. Design 1 features moderately dense arrangement of unit sensors, while Design 2 features very dense arrangement of sensors around the assumed crack propagation line. Tests aimed at identifying advantages and challenges of both designs. The two arrangements were established based on analysis of individual sensors performed in earlier research (see [26]). Each design of sensing sheet was tested two times, thus in total, four steel plates were prepared, two with the sensing sheet of Design 1 (Plates #2 and #4) and two of Design 2 (Plates #1 and #3).

**Figure 8:**
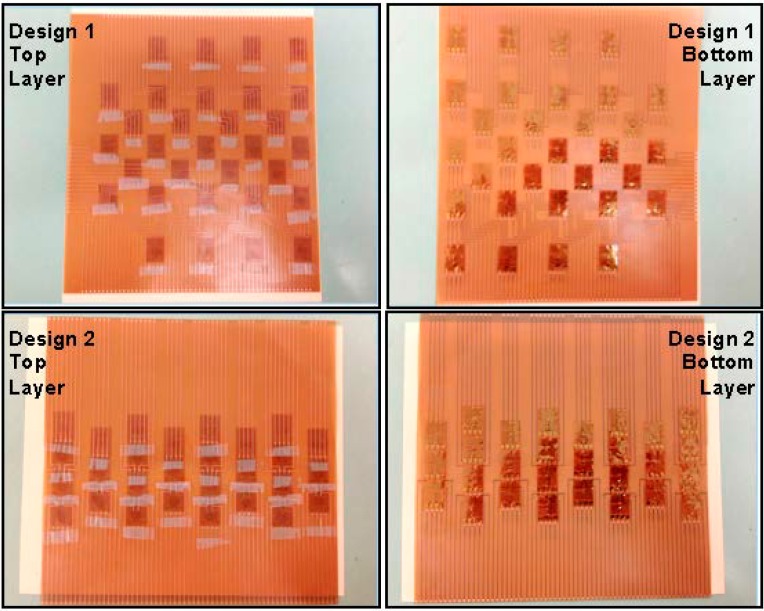
Complete assembly of top layer (left two images) and bottom layer (right two images) of two sensing sheet designs; Design 1 is shown in the two upper photographs and Design 2 is shown in the two lower photographs.

Based on the design of the sensing sheet interconnects, individual strain gauges were laminated onto the sheet as shown in Figure 8. The interconnects contained two layers of patterned electrical conductors: the top layer consisted of individual conductors, whose purpose was to guide the signals from the reading unit to individual sensors and then get signals back. The bottom layer of the interconnects was filled with hollow metal pads connected through the polyimide substrate to the conductors on the top layer. In order to avoid short circuits and direct exposure of the conductors to potential cracks in the steel plates, the strain gauges were laminated on the bottom side of the sensing sheet, using a small piece of scotch tape to maintain the relative positions. The four leads (connecting wires) of each strain gauge were passed through the corresponding pad holes and connected the sensor to the metal conducting wires on top layer by simple contact (no soldering was performed). The bottom side of the sensing sheet was then glued to the steel plate. Photographs of assembled sensing sheets with individual sensors onto the two different interconnections are shown in Figure 8. The upper two pictures show the sensing sheet of Design 1, and the lower two pictures of Design 2.

**Figure 9:**
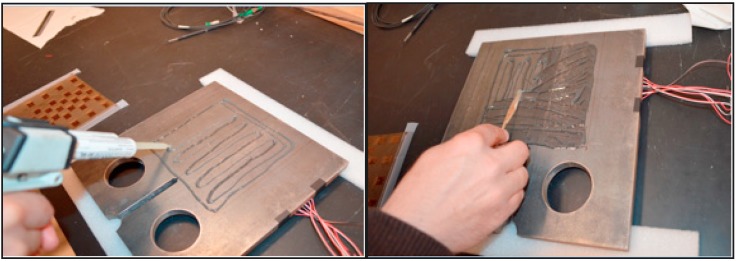
(**Left**): adhesive was applied onto the steel plate; (**Right**): adhesive was spread and excess removed.

After all individual sensors were laminated onto the sensing sheets, the latter were glued to the steel plates with the bottom layer next to steel. Both sensing sheets and steel plates were cleaned with isopropyl alcohol before the gluing. Figure 9 depicts the application of the adhesive onto a plate.

**Figure 10:**
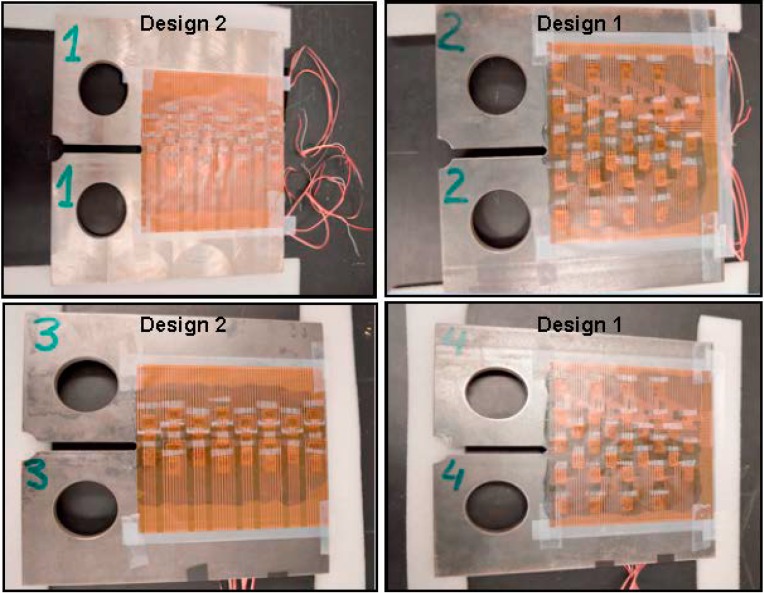
Four sensing sheet glued to the steel plates, and specimen labels are marked on the plates: Plates #2 and #4 are of Design 1, and Plates #1 and #3 of Design 2.

After the sensing sheets were laid onto the adhesive, it was important to ensure that no air bubbles were captured at the interface between the sensing sheets and the plates. The air bubbles were “pushed” out before hardening of the adhesive by pressuring the top layer of the sensing sheet with a soft cloth. Plates #2 and #4 carried sensing sheets of Design 1, and Plates #1 and #3 of Design 2. The four ready-for-test specimens are shown in Figure 10.

## 4. Cycling Fatigue Tests

The cyclic fatigue tests of steel plates were carried out in the Carleton Laboratory at Columbia University. The testing machine was used to apply cyclic load. Two fixtures (upper and lower) were used to transfer the load from the machine to the plate under the test. The plate fixtures were provided by the University of Delaware. During each test the upper fixture was immobile, while the lower fixture cyclically tensioned and compressed the plate under test, to apply fatigue load.

**Figure 11:**
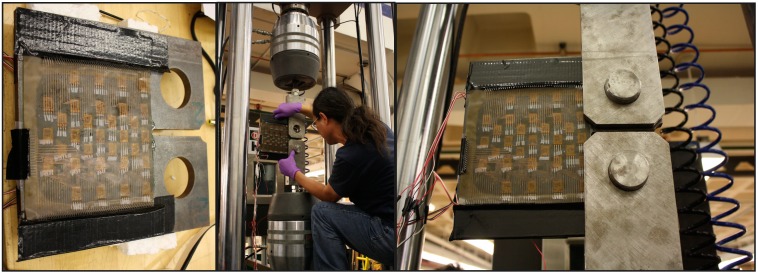
(**Left**): protection of sensing sheet connectors with insulation tape; (**Middle**): steel plate is placed into the fixtures; (**Right**): steel plates are fastened tightly with two steel pins.

**Figure 12:**
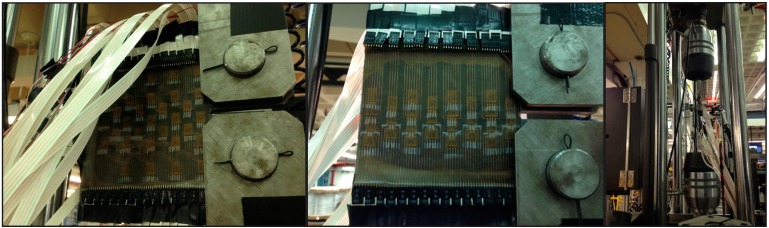
**Left**: Cable connections of sensing sheet Design 1 (for samples 2 and 4); **Middle**: Cable connections of sensing sheet Design 2 (for samples 1 and 3); **Right**: side view of cable connections and test set-up.

To prevent short-circuiting, the connecting pads of the sensing sheets were further protected by insulation tape (see Figure 11, left). Then they were attached to the fixtures, and secured tightly with pins, as shown in Figure 11, middle and right. The sensing sheet was then connected to the multi-channel reading unit using cables extensions. Photographs of the cable connections are shown in Figure 12.

**Figure 13:**
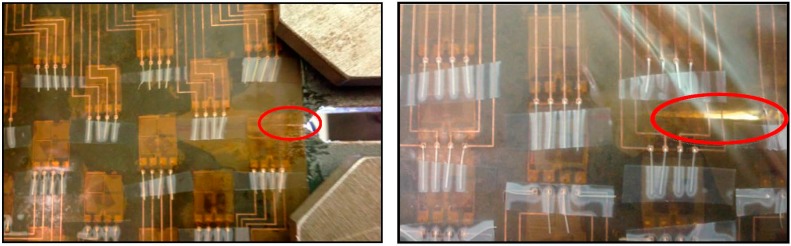
(**Left**): initial crack occurring on Plate #2 (sensing sheet Design 1); (**Right**): initial crack occurring on Plate #1 (sensing sheet Design 2).

**Figure 14:**
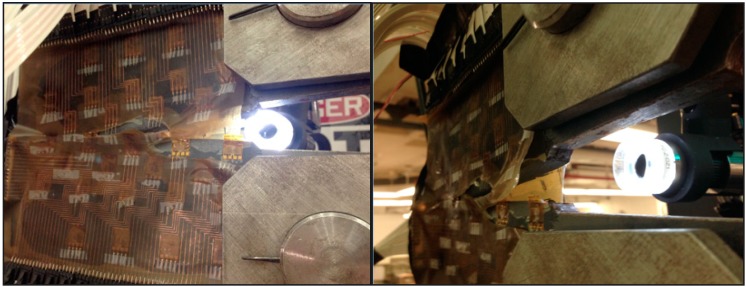
Failure of Plate #2 (sensing sheets Design 1). (**Left**): frontal view of the crack; (**Right**): side view of the crack.

The steel plates (see Figures 10) were tested based on the schedule shown in Table 1. The loading frequency varied in different tests in order to find optimal test duration, so that testing of four samples could be completed within the allocated timeframe. This variation did not affect the test results. During the cycling tests the tip of the notch zone in the steel plate was exposed to the largest stress concentration, and the initial fatigue crack was expected to occur at that location. Based on the four test observations, the initial crack appeared before 40,000 cycles were carried out. The sampling rate of individual sensors in the sensing sheet was 20 Hz. Figure 13 shows the initial cracks in Design 1 and Design 2. Rapidly increasing strain at the location of crack initiation practically served to predict the occurrence of the crack (see Section 5). The initial crack was small (length ~10 mm, width ~1 mm) and the sensing sheet was able to detect it. Under the further cycling that followed the initiation, the crack propagation was slow and it started to propagate faster only when the specimen was close to failure. The specimen was considered as failed when the vertical displacement between the two fixture pins had reached one inch (25.4 mm), and at that stage the crack ended in the area close to the middle of the plate. Figure 14 shows the failure of Plate #2.

**Table 1:** Schedule of cyclic fatigue tests.

	Plate #2 1st Period	Plate #2 2nd Period	Plate #4	Plate #3	Plate #1
No. of loading cycles	0–110,000	110,001–139,913	0–58,867	0–135,645	0–99,696
Actual load range	12–20 kip	13–24 kip	Initial: 12–25 kip	20–28 kip	20–28 kip
			Final: 14–23 kip		
Loading frequency	4 Hz	6 Hz	6 Hz	4 Hz	4 Hz

**Figure 15:**
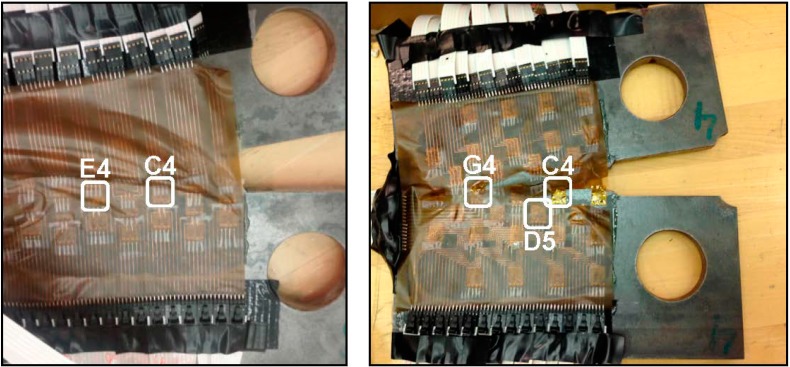
Typical failure modes of the sensing sheet under excessive crack opening; (**Left**): delamination, Plate #3 (Design 2); (**Right**): tearing, Plate #4 (Design 1).

During the failure process of the plate, the shaking caused the interconnections of the sensing sheets of Design 2 to locally delaminate; however, individual sensors were still well bonded on the steel plate. Figure 15 shows the examples of the failure of the sensing sheets under extreme crack opening. Two main identified mechanisms of the sheet failure were delamination and tearing. While tearing was the expected mode of local failure (assuming an excellent bonding of the sheet to the steel plate), the delamination of interconnects was not expected. Inspection led to conclusion that it occurred in sensing sheets of Design 2 due to imperfections in the process of lamination during which the individual sensors were connected to the interconnect only by the scotch tape. Since the sensors were densely spaced in Design 2, practically there was no solid adhesion between sensors and interconnect over an important continuous area covered by the sensors (see Figure 8 and Figure 10). This lead to delamination of the interconnections from the sensors, while sensors remained well bonded to the plate. Delamination did not happen in sensing sheet of Design 1, since the interconnections were glued directly to steel over the relatively large spaces surrounding the sensors (see Figure 8 and Figure 10). This last result is encouraging since it shows excellent bonding between the substrate and the steel and demonstrates that the issue of delamination should not be present in the real sensing sheets where the unit sensors will be directly patterned on the substrate.

Besides the interconnect delamination, another issue occurred due to simplified procedure of sensor lamination. As mentioned earlier in the text, the leads (connecting wires) of the strain gauge were simply passed through the corresponding pad holes of interconnect and connected the sensor to the metal conducting wires on top layer by simple contact, *i.e.*, with no soldering. Unfortunately, this proved to be an unreliable solution during tests: due to vibrations in the plates, the leads of some of the sensors disconnected from the interconnect and thus, they could not be measured during the tests. This issue will not exist in a real sensing sheet where the sensors will be directly patterned on the substrate along with the interconnects. In spite of these disconnections, a sufficient number of sensors was fully operational and allowed assessment of the overall performance of the sensing sheet, which also demonstrated that malfunction of several sensors does not affect the overall performance of the sheet.

It is important to note that as the crack crossed a sensor, this sensor would be damaged almost immediately. However, all the other sensors would continue functioning until either they are damaged or the sensing sheet is damaged by one of the above presented failure modes. This immediate damaging of the strain sensor was unexpected based on the findings made on concrete specimens in earlier research [26], where the sensor functioned properly until a crack of a few millimeters was formed. The main reasons for this difference are: (1) better adhesion of the sensing sheet to the steel than to the concrete and (2) degradation of concrete that occurs in the zone of the crack opening; local tensile stresses in concrete lead to degradation of the crack mouth and enable redistribution of load in the sensor, which lower its internal stresses. This phenomenon does not occur in the case of crack occurrence in steel. Hence, the response of the unit strain sensor exposed to cracks is different for steel and concrete, and the use of a different adhesive that will delaminate locally over a short length (~1–2 mm) and enable redistribution of load in the sensors (thereby lowering the stress in sensor) is advised for the installation of the sensing sheet onto the steel elements.

## 5. Measurement Results from Strain Gauge Sensing Sheets

**Figure 16:**
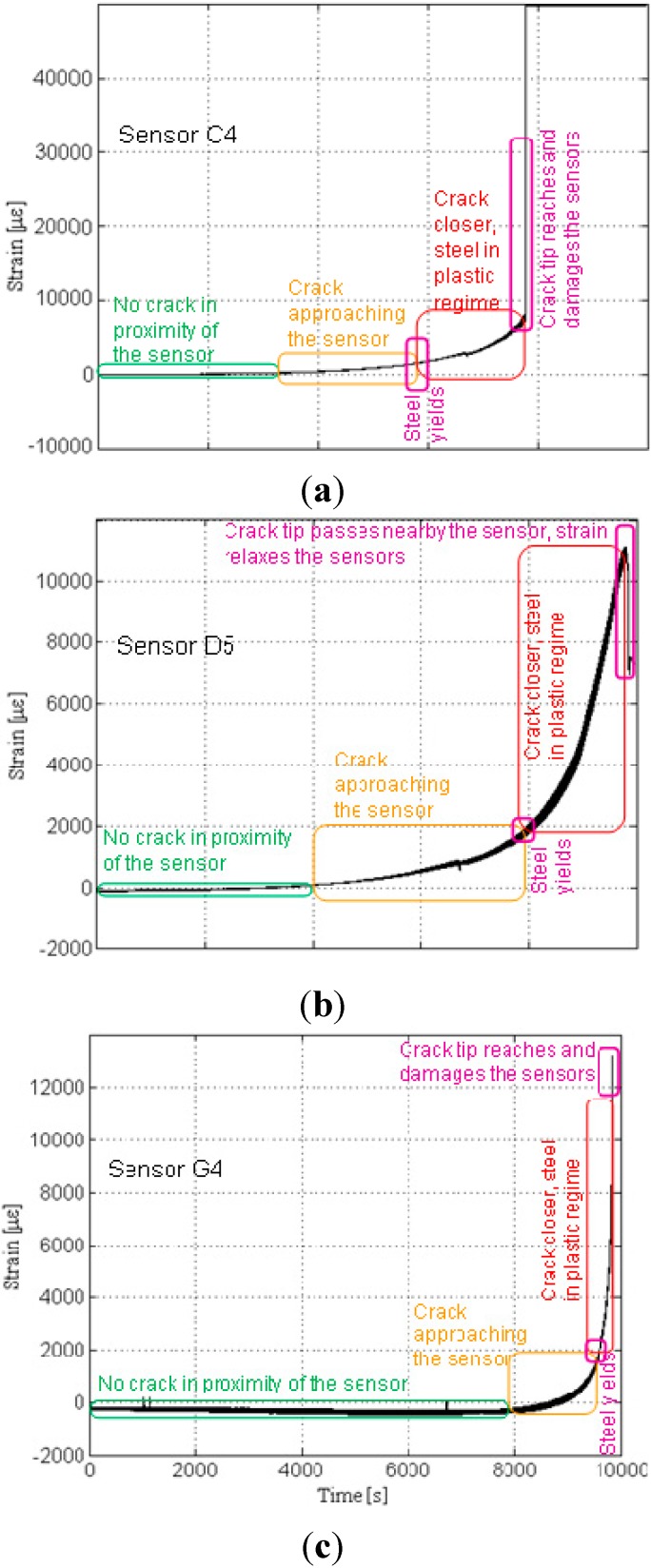
Typical sensor readings, sensors C4, D5 and G4, Plate #4 (Design 1). (**a**) Strain time history of sensor C4; (**b**) Strain time history of sensor D5; (**c**) Strain time history of sensor G4. Note: scale for strain is not the same for the three graphs.

To illustrate the general response of strain sensors from the sensing sheets, three typical individual sensors from Plate #4 (Design 1) are presented first. The selected examples are sensors with coordinates C4, D5 and G4 and these sensors are encircled in Figure 15 right. Note that crack propagates from the side ‘A’ towards side ‘I’ (*i.e.*, from right towards left, see Figure 6 and Figure 15). Thus, it first meets sensor with coordinate C and then propagates towards the sensors with coordinates D and G. Figure 16 shows the patterns of strain response depending on the crack’s distance from the sensor.

In general, the following phases were identified: (1) when the crack is far from the sensor no significant strain change is noticed; (2) strain significantly increases as the crack approaches the sensor, and this increase of the strain is an indicator of damage in the neighborhood of the sensor; (3) after the elastic limit is reached, steel yields and strain increases with a higher rate; (4) if the crack tip reaches the sensor at any stage in phases (2) or (3) the sensor is broken and damage is detected and localized (see Figure 16, top, and Figure 16, bottom); and (5) if the crack passes aside the sensor, the strain decreases significantly and damage is detected and localized based on this change (see Figure 16, middle).

Among the sensors C4, D5 and G4, the sensor C4 was the first to be crossed by the crack at approximately 7850 s from the beginning of the test; since this sensor was on the crack path it was damaged by the crack tip. The crack was detected at that location through the damaging of the sensor. The next sensor to be affected by the propagating crack was the sensor D5 at approximately 9770 s from the beginning of the test; this sensor was not on the crack path, but below it, and thus it was not damaged by the crack tip. The crack was detected at that location indirectly, as a decreasing strain trend. Finally, the sensor G4 was the last to be affected by the propagating crack, at approximately 9860 s from the beginning of the test; as it was placed on the crack propagation line, it was broken when the crack tip reached it and, like sensor C4, the damage was detected at that location through the damage of the sensor.

**Figure 17:**
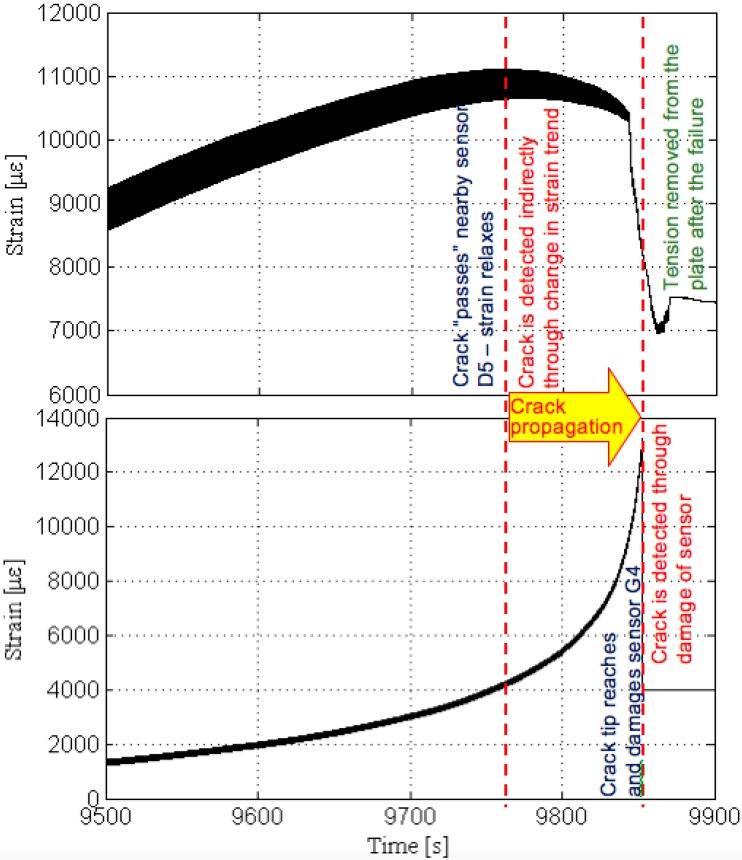
Propagation of crack from location “D” to location “G”.

In order to simplify the tracking of the crack propagation, the strain diagrams from the three sensors are presented on the same time scale in Figure 16. A close-up from diagrams of sensors D5 and G4 indicating that the crack “passes” aside sensor D5 and reaches sensor G4 is shown in Figure 17.

**Figure 18:**
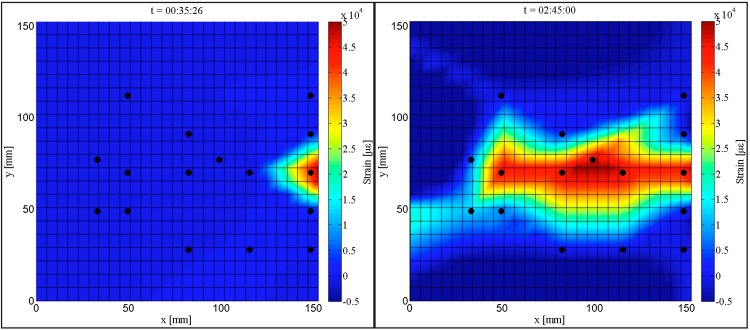
Maps of strain distributions in Plate #4 (Design 1); (**Left**): initialization of the crack (indicated in red color); (**Right**): crack extent at the end of the test.

Furthermore, based on the analysis of all operational sensors (*i.e.*, those whose leads did not disconnect from the interconnect), a time-history graph that illustrates the strain responses from the entire sensing sheet is presented. Figure 18 shows the initiation of crack and its extent at the end of the test for Plate #4 (Design 1). The coordinates of positions in the sensing sheet are shown on the boundaries and the black dots represent all operational sensors. The left image in Figure 18 shows strain distribution and the initialization of crack at time t = 35′26″ from the beginning of the test (crack is indicated by the red color, *i.e.*, very high strain value). The right image in Figure 18 shows strain distribution and the crack extent at time t = 2 h 45′00″ at the end of the test. The strain distributions presented in the maps are obtained by interpolation.

**Figure 19:**
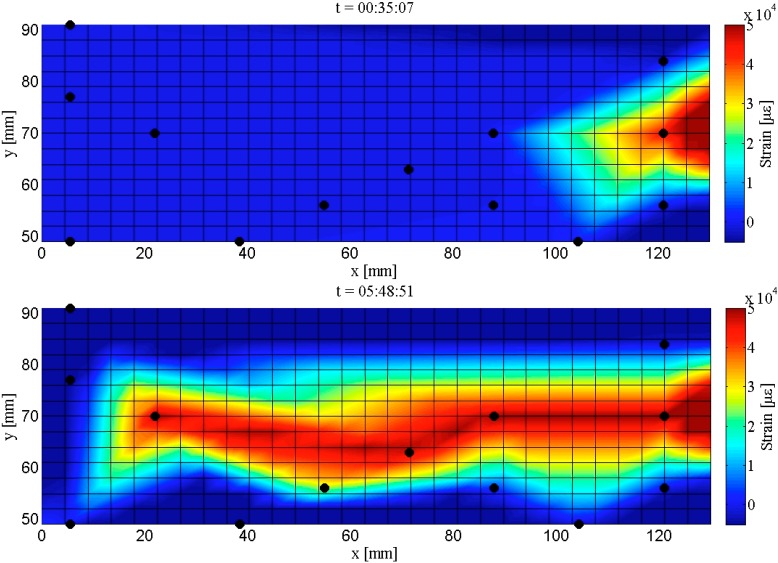
Maps of strain distributions in Plate #1 (Design 2); (**Up**): initialization of the crack (indicated in red color); (**Down**): crack extent at the end of the test.

Similar analysis has been carried out for Plate #1 (Design 2), and a time-history mapping of the strain distribution and crack propagation over the sensing sheet is shown in Figure 19. The analyzed area is narrower than for Plate #4, due to the more concentrated layout of sensors around the expected crack propagation line. The left image in Figure 18 shows the strain distribution and initialization of crack in Plate #1 at time t = 35′07″ from the beginning of the test, and the right image shows the strain distribution and extent of crack at time t = 5 h 48′51″, *i.e.*, at the end of the test. A comparison with Figure 18 shows that in Design 2, the red zone is more concentrated in the center of the sensing sheet, which better matches the visual observation and thus reflects more accurately the information on crack location and extent. This confirms that higher density of sensors provides better accuracy in crack localization and evaluation of its extent.

Due to limited space, only results from Plate #4 and Plate #1 are presented in this section, but they are representative for Design 1 and Design 2 respectively (findings from tests on Plate #2 were similar to those of Plate #4 and findings from Plate #3 were similar to those of Plate #1). While the sensing sheet of Design 2 had the benefits of a denser sensor array and more accurate damage characterization, Design 1, with less dense sensor arrangement, was also successful in damage detection and characterization. This is a very important finding, as it shows that even less dense networks could be successful in damage characterization, which may significantly simplify manufacturing of the sensing sheet, decrease its final cost, and also simplify data management and analysis. Qualitative comparison of the two designs is given in Table 2.

**Table 2:** Qualitative comparison of sensing sheet Design 1 and Design 2.

	Density of Sensors *	Manufactu-Ring Cost **	Redun-Dancy **	Accuracy in Crack Characterization **	Complexity of Data Ma-Nagement and Analysis **
Design 1	26% (21.5 sensors/dm^2^)	Low	Moderate	High	Low
Design 2	40% (33 sensors/dm^2^)	Moderate	Very high	Very high	Moderate

* Percentage of instrumented area over total area, around crack line; ** correlated with number of sensors per square decimeter.

## 6. Conclusions

This paper presents a research stage on a broader topic that involves creation of sensing sheets based on LAE. In particular, the sensing sheet concept was examined through cyclic fatigue tests on steel plates. In total, two sensing sheet designs were evaluated, and two specimens of each design were created and tested. Design 1 dealt with a less dense arrangement of sensors, while Design 2 dealt with a very dense arrangement of sensors.

In order to simplify the manufacturing and lower the costs of tests, commercial full-bridge strain gauges were laminated over the interconnects, and used in combination with a multi-channel reading unit. Simplification in the sensor lamination process raised two issues: first, a premature delamination of the interconnects during the tests in the case of Design 2, and second, the loss of contact between sensor leads and interconnects that reduced the number of fully operational sensors during the tests. However, these issues would not occur in a real sensing sheet as both the sensors and the interconnects will be patterned directly onto the substrate. Important challenge that was identified, was damage of sensor that occurred almost immediately once the crack reached the sensor. In spite of all the issues, the tests were successful and led to several important conclusions.

In general, the tests have demonstrated that the sensing sheet could perform reliable crack detection and localization, and that it could follow crack propagation in real time and evaluate the crack extent. Various phases in sensor response depending on crack position and propagation were identified. These phases indicate that damage can be detected before it reaches the sensor. It was also demonstrated that damage to individual sensors reduces the accuracy of damage characterization, but does not significantly impair the overall capability of the sensing sheet to perform damage detection. These are important findings as they prove the concept of direct sensing applied to dense arrays of strain sensors, and validate the idea of the sensing sheet.

Comparison between two designs indicated that both have advantages and challenges, and consequently optimization of the design will be a topic of future research. Future research will also include selection and optimization of adhesive that will enable sensors to survive initial cracking in steel structures (e.g., by local delamination of individual sensors). Finally, setting thresholds will be researched based on the identified phases in sensor response to propagating cracks.
